# The role of endothelin and RAS/ERK signaling in immunopathogenesis-related fibrosis in patients with systemic sclerosis: an updated review with therapeutic implications

**DOI:** 10.1186/s13075-022-02787-w

**Published:** 2022-05-13

**Authors:** Mohsen Rokni, Mina Sadeghi Shaker, Hoda Kavosi, Shahrzad Shokoofi, Mahdi Mahmoudi, Elham Farhadi

**Affiliations:** 1 Department of Immunology, School of Medicine, Tehran University of Medical Sciences, Tehran, Iran; 2 Department of Immunology, University of Social Welfare and Rehabilitation Sciences, Tehran, Iran; 3 Rheumatology Research Center, Tehran University of Medical Sciences, Tehran, Iran; 4 Inflammation Research Center, Tehran University of Medical Sciences, Tehran, Iran; 5 Rheumatology Department, Urmia University of Medical Sciences, Urmia, Iran

**Keywords:** Endothelin, Fibrosis, Ras signaling, Systemic sclerosis, TGF-β, Vasculopathy

## Abstract

Systemic sclerosis (SSc) is a disease of connective tissue with high rate of morbidity and mortality highlighted by extreme fibrosis affecting various organs such as the dermis, lungs, and heart. Until now, there is no specific cure for the fibrosis occurred in SSc disease. The SSc pathogenesis is yet unknown, but transforming growth factor beta (TGF-β), endothelin-1 (ET-1), and Ras-ERK1/2 cascade are the main factors contributing to the tissue fibrosis through extracellular matrix (ECM) accumulation. Several studies have hallmarked the association of ET-1 with or without TGF-β and Ras-ERK1/2 signaling in the development of SSc disease, vasculopathy, and fibrosis of the dermis, lungs, and several organs. Accordingly, different clinical and experimental studies have indicated the potential therapeutic role of ET-1 and Ras antagonists in these situations in SSc. In addition, ET-1 and connective tissue growth factor (CTGF) as a cofactor of the TGF-β cascade play a substantial initiative role in inducing fibrosis. Once initiated, TGF-β alone or in combination with ET-1 and CTGF can activate several kinase proteins such as the Ras-ERK1/2 pathway that serve as the fundamental factor for developing fibrosis. Furthermore, Salirasib is a synthetic small molecule that is able to inhibit all Ras forms. Therefore, it can be used as a potent therapeutic factor for fibrotic disorders. So, this review discusses the role of TGF-β/ET-1/Ras signaling and their involvement in SSc pathogenesis, particularly in its fibrotic situation.

## Introduction

Systemic sclerosis (SSc), as a connective tissue disorder, is extremely miscellaneous in its multisystem clinical manifestations, causes a high level of mortality and morbidity and follows a variably unpredictable period [1]. The etiology of the disease is unknown and is specified by ample cutaneous and visceral fibrosis caused by improper immune system activation and vascular injury [2]. Dysregulation of multiple cellular and molecular components of the innate and adaptive immune system has long been identified in SSc [3, 4]. Early fibrotic lesion seems to be the first manifestation of the disease [5]. This lesion is accompanied by prominent infiltration of bone marrow-derived immune cells including CD4^+^ T cells, monocytes/macrophages, activated B cells, plasmacytoid dendritic cells (pDCs), and mast cells [6]. Transforming growth factor-β (TGF-β) is a very strong stimulator of collagen types I, III, VI, and VII; fibronectin (FN); α-smooth muscle actin (α-SMA); and in addition reduced upregulation of matrix metalloproteinase 1 (MMP-1) by fibroblasts (those critical mediators of fibrosis) [7]. TGF-β also induces the promotion of ET-1 in endothelial cells. However, ET-1 is as well as released from fibroblasts and/or myofibroblasts and is promoted by the stimulation of TGF-β. While TGF-β and ET-1 have been remarked as major mediators involved in the SSc pathogenesis [8]. ET-1 is upregulated in both initially and end-stage SSc patients and is a critical mediator of vasculopathy. ET-1 and TGF-β are a very strongly potent vasoconstrictor that is released by vascular endothelial cells and is the main mediator of vasculopathy. Some studies indicated, in vasculopathic disorders (such as SSc), ET-1 and TGF-β are over-expressed in the endothelial cells and released from these cells, which may increase their vasoconstriction and fibrosis role [9, 10].

SSc is primarily characterized by vasculopathy which induces a broad array of changes in small and large blood vessels, perivascular infiltration of mononuclear cells, endothelial dysfunction, extracellular matrix (ECM) remodeling in the vascular wall, and the elimination of capillaries (capillary rarefaction). Recently, a novel prospect underpins the potency of the ET-1/H-Ras/ERK1/2 pathway in the pathogenesis of vasculopathy. For instance, the formation of fibrosis, the vasculitis, and the production of autoantibodies in SSc patients are shown to be affected by the circulating levels of the TGF-β- and/or ET-1-related markers [10].

Furthermore, inflammation maximizes the likelihood of the constant occurrence of processes leading to fibrosis. Indeed, an inflammatory infiltrate is a characteristic of the initial phases of fibrosis. Inflammatory cells can modify fibroblast metabolism which may profoundly affect the ECM production and collagen synthesis by direct cell-cell interactions and/or by secreting soluble products such as TGF-β, connective tissue growth factor (CTGF), interleukin (IL)-6, altered Ras signaling and altered responses to ET-1 [11]. So, the recognition of disease mechanisms in order to target both fibrotic and vascular events and pathways that interconnect vasculopathy and organ fibrosis is vital [12, 13]. This review discusses about the current knowledge about the role of the ET-1/H-Ras/ERK1/2 pathway in the fibrosis and vasculopathy, with particular emphasis on the regulation of this pathway in the fibrotic microenvironment, the molecular mechanisms, and signaling involved in its pro-fibrotic actions, as well as the contribution of ET-1/H-Ras/ERK1/2 pathway in SSc and focusing on potential therapeutic implication.

### Vasculopathy, fibrosis, and organ failure in systemic sclerosis

**Fig. 1:**
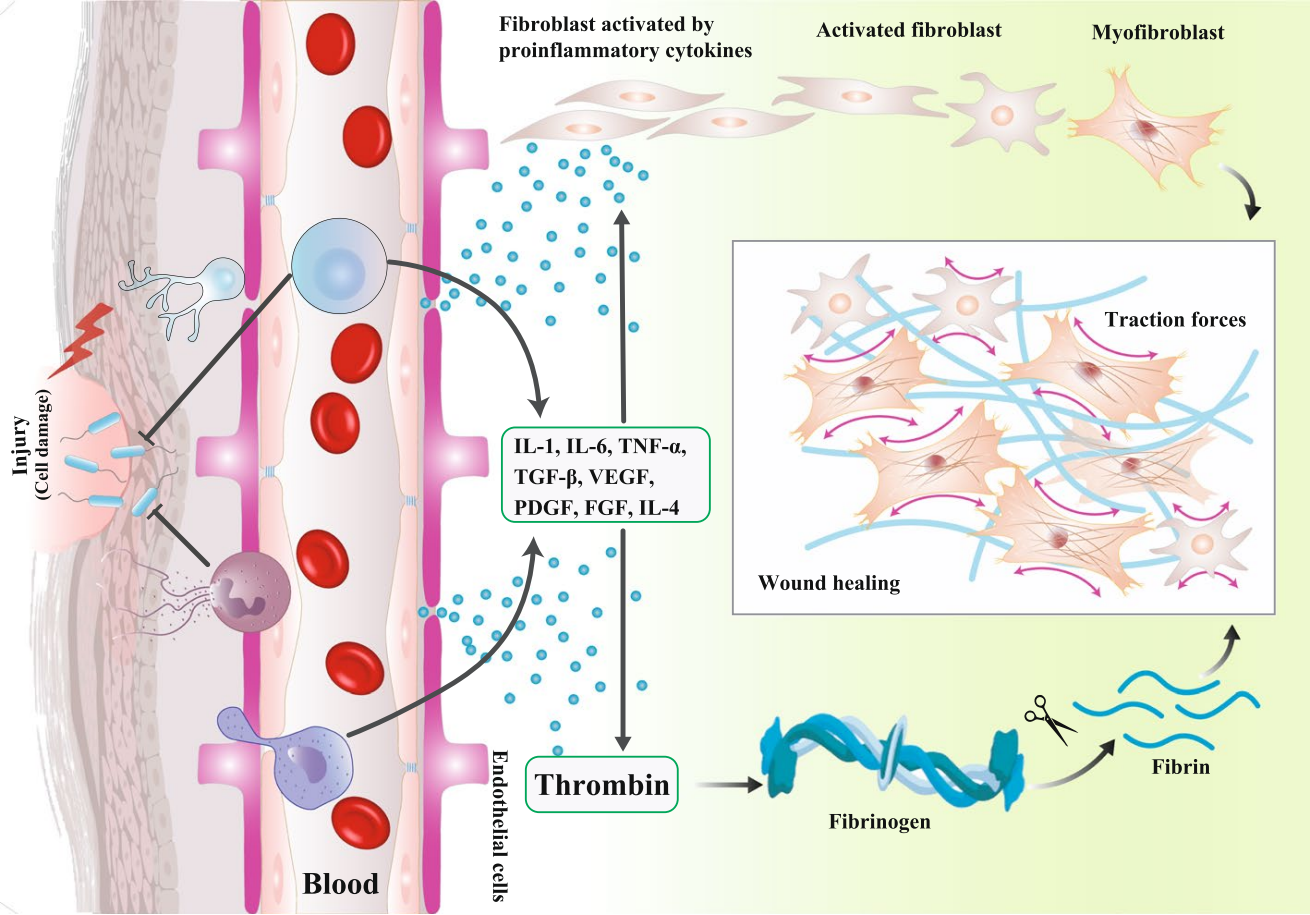
Wound healing results from a well-coordinated series of events divided into four overlapping phases: hemostasis, inflammation, proliferation/matrix deposition, and tissue remodeling. Neutrophils and macrophages are particularly important in mediating this process, though T cells and platelets also play key roles. Meanwhile, immune cells themselves release factors such as chemokines and cytokines to amplify inflammatory responses. Next, the inflammatory mediators, such as TGF-β, PDGF, IL-13, IL-6, and IL-4, induce the limited activation and proliferation of myofibroblasts from fibroblasts. In addition to resident fibroblasts, myofibroblasts are derived from multiple cells, including fibrocytes, epithelial cells via EMT, endothelial cells via EndMT, pericyte cells, and smooth muscle cells related to the blood vessels. Activated myofibroblasts migrate to injury sites and stimulate wound closure through cell-generated traction force

Fibrosis is defined as pathological outcomes of normal wound healing (Fig. 1) [14] and the main hallmark of SSc [15]. The vascular abnormalities (vasculopathy) in SSc may not need inflammatory events and would better be characterized as an intimal hyperplasia in the capillaries, sinusoids, and the tiny blood vessels, without vasculitis [16]. The first vasculopathy signs include increased vessel permeability, dysregulated control of vessel tone, and endothelial cell injury. In addition, the endothelial cell injury leads to the overexpression of von Willebrand factor (VWF), ET-1, and increased circulating survived or apoptotic endothelial cells [15].

**Fig. 2:**
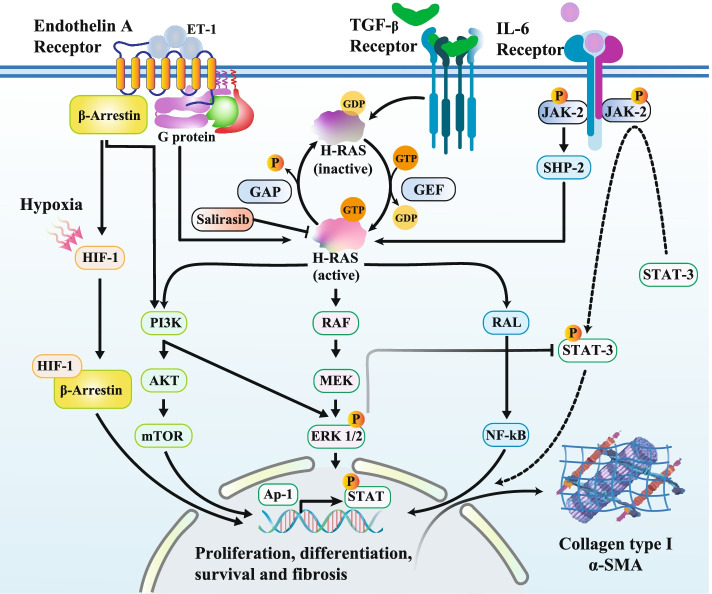
Schematic representation of a model for the signaling pathway involved in ET-1, TGF-β and IL-6/Ras-induced ERK1/2 activation, thus increasing fibrosis produce in systemic sclerosis fibroblasts. Salirasib is a synthetic small molecule that is able to inhibit all Ras forms

Many molecules have a remarkable role in the vasculopathy and pathological fibrosis in SSc including TGF-β, GTPase H-Ras, ET-1, IL-6, and numerous biologically active substances [17–19]. IL-6 could enhance collagen production in fibroblast cells, through direct mechanisms (JAK2-STAT1/3) and enhances of TGF-β signaling pathway (Indirect) [19]. The dysregulation of the TGF-β-ET-1-Ras signaling pathway seems to be one of the most involved in the pathogenesis of SSc, and this issue is confirmed by the high expression of *TGFB*-*ET1*-*RAS* genes in skin biopsies which is positively associated with disease severity [20]. It links fibrosis and vasculopathy, and can induce the myofibroblast phenotype. Accordingly, the number of α-SMA-positive myofibroblasts is enhanced and the dermis becomes atrophic. The thickness of the epidermis gets diminution and small-sized blood vessels become virtually disappeared. Vascular rarefaction leads to tissue hypoxia and induces the hypoxia-inducible factor-1 (HIF-1). Activation of fibroblast and fibrosis progression is induced following hypoxia as an independently potent stimulus (Fig. 2). The resulting fibrosis leads to the destruction of the physiological structure of tissues and, thus, causes of functional impairment in the affected organs in the early phase of SSc [21]. Myofibroblast elimination by apoptosis is supposedly a crucial mechanism in this resolution phase, where failure in this process can contribute to fibrosis development [22]. In this respect, ET-1/TGF-β and its signaling pathway (Ras-MEK-ERK1/2) have been considered to enhance myofibroblast resistance to apoptosis, thereby contributing to the persistence of the fibrotic response.

Finally, dysfunction of affected organs in SSc is the result of progressive replacement of healthy contexture architecture by collagen-rich ECM. The fibrotic process occurs most prominently in the dermis, lungs, heart, and gastrointestinal tract and also widespread perivascular fibrosis occurs [23].

### Endothelin-1, interleukin-6 and TGF-β synergistic role in pathogenesis of systemic sclerosis

#### Molecular biology of the endothelin system

The ET family contains three isoforms, namely ET-1, ET-2, and ET-3, as the products of various genes. Although ET-2 is different from ET-1 only in two amino acid residues and ET-3 is different from ET-1 in the six amino acid residues, they are considered as different gene products with tissue-specific expression [24]. They are produced as precursor proteins, pre-pro-ETs (about 200 amino acids), and then cleaved by an endo-peptidase so that they can give form to an inactive precursor of about 37–41 amino acids and big ETs. Finally, the conversion of big ETs to the mature 21 amino acid peptides is done by a family of ET-converting enzymes [25]. The level of pre-pro-ET-1 is regulated predominantly by the multiple transcription factors such as activator protein 1 (Ap-1), nuclear factor kappa B (NF-κB), forkhead-box protein O1 (FOXO1), HIF-1α, and GATA2 [26, 27]. ET-1 is produced normally by endothelial cells, epithelial cells, mast cells (in asthma), macrophages, polymorphonuclear (PMN) leukocytes, cardiomyocytes, and fibroblasts [18]. All ET subtypes act through G protein-coupled receptors (GPCRs) such as ET_A_receptor (ET_A_R) and ET_B_R. The ET_B_R has two subtypes, namely ET_B1_ and ET_B2_. While the ET_B_R is present on both smooth muscle and vascular endothelial cells, the ET_A_R can be found predominantly on smooth muscle cells (SMCs) [28]. Given the high affinity of ET-1, a long-lasting vasoconstriction effect is mediated primarily through the ET_A_R [29]. On the other hand, increased expression of endothelin and their receptors cause vasodilatation. ET_B_R signaling in endothelial cells by nitric oxide (NO) and prostacyclin production can stimulate vasodilation [30]. ET-1 as a cofactor of the TGF-β cascade plays the main role in fibrosis through induction of collagen production and differentiation of fibroblasts into myofibroblasts, also contributes to vascular impairment in SSc [31]. The plasma concentration of ET-1 is increased, and ET-1R is overexpressed in the pre-sclerotic and early diffuse skin lesions in SSc patients [32]. Besides, a high plasma level of ET-1 is associated with the number of digital ulcers in SSc patients [33]. The pharmaceutical dual ET_A_/ET_B_ receptor antagonist or selective ET_A_R inhibitors reduces the mortality rate in the pulmonary arterial hypertension (PAH) patients [34]. While ET_A_R signaling is essential in skin fibroblast activation, the role of ET_B_R signaling in fibrogenesis is ambiguous.

#### Endothelin-1, TGF-β, and their signaling pathway in fibrosis and vasculopathy

ET-1 and NO acts against each other to maintain vascular homeostasis and make a balance between vasoconstriction and vasodilation [35]. The overexpression of ET-1, specifically in endothelial cells, could lead to systemic hypertension with altered vascular reactivity in an experimental model [36]. It has been reported the severity of fibrosis is associated with the level of local ET-1 [37, 38]. In vitro treatment of fibroblasts by ET-1 leads to the enhanced production of collagen types I, III, and FN and reduced expression of MMP-1 (critical mediators of fibrosis) via ET_A_R and ET_B_R [39]. ET-1 overexpression in micro-vascular endothelial cells (MVECs) of the upper skin is accompanied by an upregulation number of ET-1_B_R in the SSc dermis and lungs. So, due to the diverse biological functions of ET-1, it has been suggested that ET-1 can link between the veins abnormalities and fibrotic process in SSc [16]. ET-1 can also induce CTGF production and exert its fibrotic effects by triggering a tissue factor (CD142 or factor III)/thrombin amplification loop. Furthermore, ET-1 involves fibrosis through induction of the endothelial-mesenchymal transition (EndMT) process [40].

One of the main characteristics of fibrotic disease including SSc is the presence of abnormal α-SMA-positive myofibroblasts. ET-1 can induce the expression of α-SMA and other contractility-related molecules via ET_A_R and a Rac/PI3K/Akt-dependent pathway [20]. In addition, it has been reported that the expression of α-SMA, collagen, and the CTGF in SSc fibroblasts is inhibited by the ETR blocker and bosentan [41].

One of the seven-transmembrane domain receptors (also known as GPCR_S_) is the ET-1R that its binding to ET-1 leads to the activation of G-protein-dependent primary effectors such as phospholipase Cβ (PLCβ). PLCβ cleaves phosphatidylinositol-4, 5-bisphosphate (PIP2) into diacylglycerol (DAG) and inositol triphosphate (IP3), leading to calcium effluxion from endoplasmic reticulum (ER) and protein kinase C (PKC) activation, and activation of ERK1/2. Meanwhile, activation of ET-1R leads to Ras-Raf-MEK activation, converging on ERK1/2 signaling [27]. ET-1 can induce PKB (also known as Akt) activation in myofibroblasts, vascular endothelial cells, cardiomyocytes, and SMCs. PKB activation often causes changes in the expression of genes that are involved in the cell survival [20, 42]. Notably, ET-1R also can activate ERK1/2 and PI3K/Akt/β-catenin pathway via β-arrestin-1. On the other hand, β-arrestin-1 induces VEGF expression through the activation of HIF-1α (Fig. 2) [27].

TGF-β plays an essential role in the early phase of SSc disease fibrosis, and ET-1 acts as an important downstream regulator or cofactor in the fibrosis process by TGF-β [41]. TGF-β signals through Ser/Thr kinase receptors TGF-βRI [activin receptor-like kinase 5 (ALK5)] and TGF-βRII. In the canonical pathway, ligand-activated TGF-βRI/RII induces the phosphorylation of receptor-regulated Smad (R-Smad), Smad2/3. The phosphorylated Smad2/3 then forms a complex with a co-mediator Smad (Smad4) which is transported to the nucleus and binds to the gene promoters. The p38-MAP kinase and PI3K pathways are also activated by TGF-β [43]. Furthermore, it has been reported that in the fibrotic lung fibroblasts, TGF-β1 can activate ET-1 signaling through type I receptor c-Jun N-terminal kinase (c-JNK)-/Ap1-dependent and ALK5-dependent pathways but independent of Smad proteins [41]. Both TGF-β/ALK5-dependent and TGF-β/ALK5-independent mechanism prompts the upregulation of ET-1 in SSc lung fibroblasts. Also, ET-1 can induce JNK activation and α-SMA upregulation through TAK1, so dysregulated constitutive JNK phosphorylation contributes to the increased ET-1 production in the fibrotic lesion [44]. Endothelial cells produce and release TGF-β that can induce differentiation and activation of SMCs. Mice with lacking TGF-β signaling molecules indicted defects in the vasculogenesis or blood vessel structure that is demonstrative of a defect in endothelial progenitor cells and impaired development of SMC [45]. Some studies have indicated that TGF-β can stimulate the EndoMT process. TGF-β can also induce the release of VEGF from SMCs through the p38-MAPK pathway [46]. With regard to the effect of TGF-β on ET-1 promoter activity through the ALK5/Smad3 pathway, the inhibition of ALK5 can block the anti-angiogenic TGF-β effect [47, 48].

The interruption of the constitutive c-JNK activation in fibrotic fibroblasts by dual ET_A_/ET_B_R antagonist of bosentan provides the evidence of an autocrine endothelin loop of ET-1 in its fibrotic effects [41]. Based on these results, the balance between Smads and ET-1 signaling is disrupted in fibroblasts of SSc patients. Besides, ET-1 may also mediate a pro-fibrotic phenotype of SSc fibroblasts which is less dependent on the TGF-β/Smad signaling pathway. So, the Smad-dependent pathway may not be the principal signaling in ET-1-induced fibrosis in the fibroblasts of SSc patients [49] and possibly fibrosis caused by the PI3K/Akt/ERK pathway [22]. Furthermore, blockade of ET_A_R or ET_B_R by neutralizing antibodies (NAb) or antagonists leads to lower TGF-β secretion by fibroblasts and attenuates the fibrotic phenotype [50]. Consequently, although TGF-β plays a role in the primary stage, ET-1 and/or CTGF as cofactors of TGF-β are involved in the maintenance of the fibrosis process. Thus, prospective clinical treatments can consider the benefits of both anti-TGF-β and anti-ET-1/CTGF products to target incurable diseases.

#### RAS/RAF and their signaling pathway in fibrosis and vasculopathy

All family members of Ras protein belong to a protein category named small GTPase and are involved in transferring signals within the cells (cellular signal transduction) [51]. Ras is the prototypical member of the Ras superfamilies which are involved in three-dimensional structures and control various cell behaviors. The three human *RAS* genes are encoding similar proteins created from chains of 188 to 189 amino acids [52, 53]. The Ras superfamily of oncogenes includes *HRAS*, *KRAS*, and *NRAS*, the last of which produces the K-Ras4A and K-Ras4B isoforms from alternative splicing [53].

Ras proteins function as binary molecular switches which can control intracellular signaling networks. Ras-regulated pathways control the main processes such as cytoskeletal integrity as well as proliferation, differentiation, adhesion, apoptosis, fibrosis and migration of cells [54]. When Ras is “switched on” by incoming signals, other genes involved in differentiation, fibrosis, survival, and other mechanisms will be subsequently switched on. Mutations in *RAS* genes can result in permanently activated Ras proteins. This consequently can cause unintended and hyperactive signaling, even when incoming signals are absent [55].

The *HRAS* gene is the most prevalent oncogenes in human cancer; the mutations which lead to the permanent activation of H-Ras are found in 20 to 25% of all human tumors [53] and some fibrotic diseases [56] such as SSc. It has also been reported that Ras is involved in the modulation of the immune response and influences the upregulation of major histocompatibility complex (MHC) molecules, cytokine release, antigen processing, regulation of T cell receptors (TCR), and growth mediators [57]. Previous studies have also documented that there is an enhanced Ras expression in inflammatory disorders such as systemic lupus erythematosus (SLE), nephritis, and neuritis [58–60]. For this reason, Ras antagonists are studied as a cure for disorders with Ras overexpression [56].

Teresa Grande et al. have assessed the contribution of Ras protein to renal fibrosis using the well-established mouse model of unilateral ureteral obstruction. Lower levels of both epithelial-mesenchymal transition (EMT) and proliferation of fibroblast induction were observed 15 days after obstruction in obstructed kidneys of *H-ras* knockout mice and fibroblast cell lines. Intriguingly, FN, collagen type I accumulation, overall interstitial fibrosis, and the population of myofibroblast were also lesser in the mice of *H-ras* knockout compared to the wild-type ones. According to the results of this study, lesser levels of activated Akt were observed in the kidneys and cultured fibroblast cell lines of the *H-ras* knockout mice [61].

Some studies have reported a direct role of the PI3K/Akt and Ras-MEK-ERK1/2 signaling pathways in the fibrosis enhancement in diverse tissues and used their inhibitors as potential antifibrotic medications. Salirasib (S-trans,trans-farnesylthiosalicylic acid/FTS) is a synthetic small molecule that is able to inhibit all Ras forms. By contrast, farnesyltransferase antagonists fail to block H-Ras function that is due to alternative membrane-binding mechanisms [62]. It has been shown that salirasib can inhibit progression and even regress hepatic fibrosis in both humans and rats [63]. So, it can be used as a potent therapeutic factor for fibrotic disorders such as SSc.

#### Endothelin-1 and TGF-β activate MAPKs kinase through the RAS/RAF/ERK-dependent pathway

ET-1 has the ability to activate MAPK family such as extracellular signal-regulated kinases 1 and 2 (ERK1/2), p38-MAPK, and c-JNKs in fibroblasts, cardiomyocytes, and vascular SMCs [40]. ERK1/2 has been reported to be involved mainly in the proliferation of fibroblasts, and the p38-MAPK contributes to ECM production by fibroblasts [64]. Proto-oncogene tyrosine-protein kinase Src (c-Src) is an upstream regulator of ET-1 in SMCs. Several studies have described the role of c-Src and Ras in the ET-1-induced phosphorylation of ERK1/2, c-JNK, and p38 MAPK and the regulation of early growth response protein-1 (Egr-1) expression in SMCs [40, 65, 66]. Vascular remodeling requires the presence of Egr-1 as a transcription factor. Any changes in extracellular stimuli cause the overexpression of Egr-1 in the endothelial cells which leads to the upregulation of several genes including PDGF, fibroblast growth factor (FGF), cyclin D1, IL-2, and TGF-β. So, vascular regeneration and the progression of vascular lesions are highly dependent on Egr-1 [67]. With regard to MAPK activation and induction of Egr-1 expression through c-Src and ET-1 [65], treatment of the SMCs with PP2, a selective inhibitor for Src-family kinases [68, 69], reduces the activation of the three MAPKs and the expression of Egr-1 [65]. Besides, ET-1 can activate c-Raf (rapidly accelerated fibrosarcoma), an effector of Ras, in SMCs. So, the knockdown of Ras by siRNA suppressed ET-1-induced MAPK activation [66]. These results indicate that c-Src and H-Ras plays a critical role in mediating ET-1-induced ERK1/2-MAPK activation and Egr-1 expression in SMCs [65, 66]. Some studies report that Egr-1 is highly expressed in the dermis fibrotic tissue and fibroblasts in SSc patients. This issue demonstrated “an Egr-1-responsive fibrotic genes signature” containing many genes involved in cell proliferation and differentiation, ET-1/TGF-β /Ras/ERK1/2 signaling pathway, wound healing, ECM production, and vasculogenesis. This gene signature did not correspond with the severity of SSc disease [70].

It has been shown that H-Ras-GTPases are expressed during fibrosis and play pivotal roles in regulating both proliferation of cell and TGF-β induced EMT [61, 71]. The Ras-induced ERK1/2 pathway (Raf/MEK/ERK) is typically activated by ET-1 [66], TGF-β [72], and growth factors through their receptors, which can mobilize adaptor proteins such as Shc and Grb2, and guanine nucleotide exchange factors (GEFs) like Sos. The ET-1R, TGF-βRI/RII, and receptor tyrosine kinases (RTKs)-mediated activation of a specific GEF can activate the small GTPase H-Ras through GTP loading (Fig. 2) [73, 74].

The relationship between activated Ras and Raf family proteins (B- and C-Raf), through sequential phosphorylation, leads to MEK and ERK1/2 activation [74, 75]. Although the Ser/Tyr kinase activity of ALK5s are lower than that of RTKs, the phosphorylation of ShcA (through the TGF-β receptor) triggers the association of Grb2 to Sos, leading to the activation of the Ras/ERK pathway [73]. The impacts of the Ras/ERK cascade on TGF-β1-induced EMT vary among various cell types [76]. Many studies have indicated that active Ras and the ERK1/2 cascade are needed for TGF-β1-induced EMT in keratinocytes of humans [72, 77, 78]. Moreover, the Ras/ERK pathway can interfere with TGF-β1-Smad signaling-mediated EMT in primary cultured alveolar epithelial cells and their cell lines [79]. Controversially, Watanabe-Takano et al. have shown that the Ras-ERK1/2 pathway negatively regulates TGF-β1-induced EMT in pulmonary fibrosis, and DA-Raf1 plays a pivotal role in EMT, as a dominant agonist of the Ras-ERK1/2 pathway. The results of this study imply that TGF-β1-induced Smad2/3 signaling is necessary for EMT (from RLE cells) to myofibroblasts expressing α-SMA, collagen type I, and FN. Furthermore, activated ERK1/2 might interfere with the nuclear translocation of Smad2/3, which is needed to induce EMT in RLE-6TN cells [72]. In addition to the canonical signaling, TGF-β induces non-canonical signaling, including the Ras-ERK1/2 pathway, c-JNK/p38 in MAPK, and PI3K-Akt signaling cascades [80]. These pathways finally activate the mammalian target of rapamycin (mTOR), thereby facilitating protein synthesis, cell migration, and proliferation and develop persistent fibrosis [81]. Furthermore, activation of PI3K and TGF-β/Smad3 signaling in SSc fibroblasts leads to *COL1A2* mRNA stabilization [82]. Finally, wide-ranging biologic effects of TGF-β can be produced through the combination or cross-talk of these non-Smad signaling pathways or between Smad and non-Smad signaling [75].

TGF-β/TGF-βR system induces the ET-1 promoter through JNK activation, leading to ET-1 overexpression. These processes result in increased expression of CTGF and other pro-fibrotic proteins [83]. Using genome-wide expression array analysis, Xu et al. have shown that the treatment of normal lung fibroblasts with ET-1 induces the expression of CTGF directly [84]. They have explained that ET-1 induces the MEK/ERK-MAP kinase pathway in fibroblasts. Additionally, blockade of the MEK-ERK1/2 kinase pathway with U0126, a highly selective inhibitors of both MEK1 and MEK2, a type of MAPK/ERK kinase [84, 85] and MK2206 (an Akt inhibitor) [86] inhibits the ET-1 effects on the expression of matrix-associated mRNAs and the CTGF protein. So, ET-1 could induce a program of matrix synthesis and CTGF from MEK/ERK-MAPK cascade in the fibroblasts of the lung and plays an essential role in connective tissue deposition during wound healing, fibrosis of pulmonary, and vasculopathy [84].

**Fig. 3:**
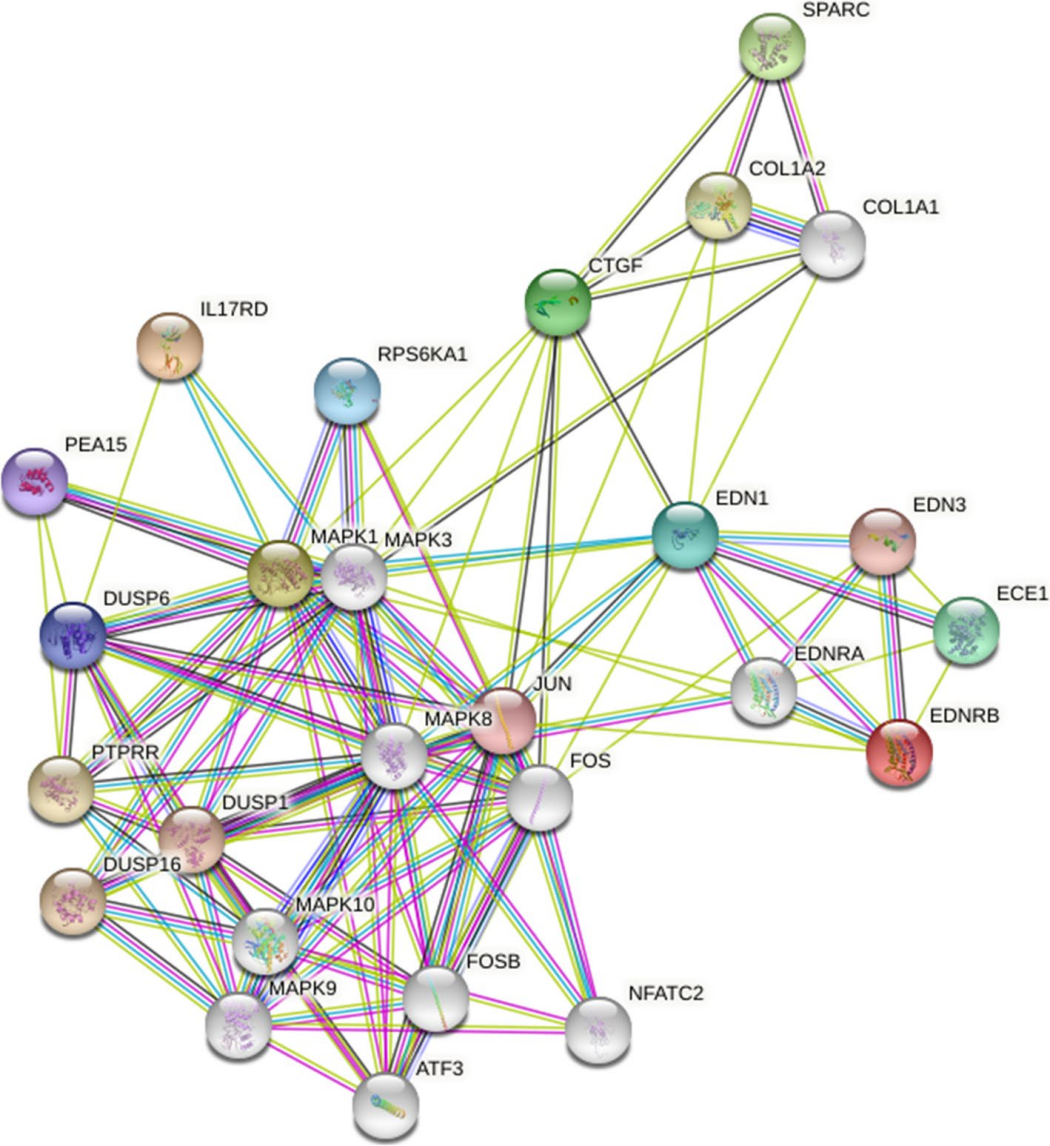
The relationship between different proteins related with fibrotic disorders

Based on discussed before, ET-1 and CTGF as cofactors of the TGF-β cascade play a substantial initiative role in inducing fibrosis. Once initiated, TGF-β alone or in combination with ET-1 and CTGF can activate several kinase proteins such as the Ras-ERK1/2 pathway that employs as the main factor for developing fibrosis. As a result, multi-step theories of fibrosis mechanism in SSc patients have been proposed: TGF-β begins fibrosis, and ET-1/CTGF acts in the maintenance process, and Ras-MEK-ERK1/2 pathways amplify persistent it (Fig. 3).

#### The role of interleukin-6, TGF-β, and RAS signaling in the fibrogenic phenotype

ET-1 also induces the pro-inflammatory cytokines expression, such as IL-6 in cultured human smooth muscle and macrophage cells. On other hand, the fibrotic marker production (CTGF, α-SMA, and collagen type I) by human fibroblasts in vitro can also be induced by IL-6 even though TGF-β is known to be the primary pro-fibrotic factor [87]. Thus, IL-6 and TGF-β may construct a self-sustaining loop leading to fibrosis that can be interrupted by the inhibition of either or both cytokines. The major downstream signaling element for IL-6 and STAT3 has been indicated to be a significant TGF-β-dependent molecular checkpoint of fibrosis in SSc patients [88]. However, fibroblasts cultured from lesion dermal of SSc patients have increased phosphorylated STAT3 forms. JAK2 inhibition, that located in upstream of STAT3 can decrease collagen levels in SSc fibroblast cells and/or in the mice models. Thus, it has been proposed that JAKs play the main role in fibrosis. Further evidence comes from the finding that a blockade of STAT3 can reduce both upregulation of collagen type I and proliferation genes (such as c-Myc) that are essential in controlling fibrosis-related genes [89]. The other downstream signaling pathways of IL-6, the Ras-ERK1/2 and PI3K-Akt cascades are derived from the glycoprotein 130 (gp130), a subunit of IL-6 receptor (IL-6R) (Fig. 2) [90, 91]. Some studies have indicated the interaction network between the Ras and the IL-6/STAT3 cascade through overexpression of IL-6 [92, 93]. With regard to the Ras/p38-MAPK pathway role downstream of both TGF-β and IL-6 [94], blocking the Ras signaling pathway may represent a novel approach to controlling both the TGF-β pathway and the inflammatory pathway in SSc [88].

Interestingly, recent reports have suggested that non-Smad pathways of TGF-β and IL-11 signaling operate through ERK1/2 to reason fibrosis and this effect is dependent on IL-11, while STAT3 phosphorylation was restricted. This data show that IL-11 can ERK1/2 activated alone or in combination with TGF-β stimulation promotes fibrosis in skin fibroblasts of SSc patients. As a result, it is improbable that STAT3 phosphorylation is directly associated with the effects of IL-11and IL-6 on fibrosis signature, although indirect systems with ERK1/2 could play an important role [95].

### Targeting the fibrotic signaling pathways: pharmacological options

Motivated by huge clinical burdens, continuous intense studies have been conducted on medication targeting fibrosis, many of which have led to clinical trials [96]. Due to the strong relationship between fibrosis and inflammation, more efforts have been devoted to drugs that may affect inflammatory signaling in the past years (Table 1).

**Table 1:** In vitro/in vivo studies and clinical trials on medications affected fibrotic disorder-affected fibrosis genes

Product name	Description and fibrosis-affected mechanism	Status of clinical development for fibrotic disorders (in vivo/in vitro studies)	NCT number for fibrosis disorders	Proposed dose for fibrotic disorders	Side effects
**Imatinib** (Gleevec, Glivec STI-571, CGP 57148)	Specific tyrosine kinase receptor inhibitor, e.g., Imatinib mesylate binds to the amino acids of the BCR/ABL tyrosine kinase ATP binding site	Phase II, III clinical trial nephrogenic systemic fibrosis Phase II and III clinical trial IPF and pulmonary fibrosis Phase II clinical trial SSc	NCT00677092 NCT00981942 NCT00131274 NCT00613171	600 mg orally, once per day for approximately 2 years	Include vomiting, diarrhea, muscle pain, headache, rash, fluid retention, gastrointestinal bleeding, bone marrow suppression, liver problems, and heart failure
**Ambrisentan** (Letairis)	A endothelin receptor antagonist and is selective for the type ET_A_ receptor	Phase II, III clinical trial idiopathic pulmonary fibrosis Clinical study SSc-PAH	NCT00768300 NCT00879229 NCT03074149 NCT03827200	5 or10 mg tablet administered orally once daily for 4 weeks	Stuffy nose, runny nose, sinus pain, headache, abdominal/stomach pain, vomiting, constipation, and sore throat
**Sirolimus** (Rapamycin)	A macrolide compound that is used to mTOR inhibition	Phase II, III clinical trial idiopathic pulmonary fibrosis Phase I clinical trial SSc	NCT03502902 NCT00241189	6 mg daily for one year	Bleeding from the gums or nose, bone pain confusion, blurred vision severe vomiting
**MMI-0100**	A novel cell-permeant peptide inhibitor of MAPKAP kinase II (MK2)	Clinical study and trial acute inflammatory response in fibrosis	^a^NCT02515396 (Unacceptable safety)	Not available	Hepatotoxicity, cardiotoxicity, and undisclosed CNS toxicity
**Salirasib** (Trans-farnesylthiosalicylic acid [FTS])	A salicylic acid that preventing activation of Ras signaling	Hepatic fibrosis/HSC	Not available	Not available	Habituation, excitation, insomnia, depression, premature closure of epiphyses
**Bosentan** (Tracleer)	A dual endothelin receptor antagonist medication	Phase I, II, III, IV clinical trial in systemic sclerosis and digital ulcers	NCT00226889 NCT01241383 NCT01395732 NCT02798055 ^a^NCT01836263	62.5 mg twice daily (b.i.d) for 4 weeks and then 125 mg b.i.d for 20 weeks	Flushing, upset stomach, fatigue, tiredness, headache, swelling of the feet/ankles/legs, and itching
**PP2**	A selective inhibitor for Src-family kinases (PI3K/Akt signaling)	Not available	Not available	Not available	Tiredness, changes in vaginal bleeding, breast tenderness
**U0126**	A highly selective inhibitor of both MEK1/2, a type of MAPK/ERK kinase	Clinical study in pulmonary fibrosis	Not available	Not available	Not available
**MK2206**	A inhibitor of pan-Akt	Not available	Not available	Not available	Not available
**Tocilizumab** (Actemra, Atlizumab)	A humanized monoclonal antibody against the interleukin-6 receptor (IL-6R)	Phase III clinical trial in systemic sclerosis	NCT02453256 NCT01532869 ^a^NCT03610217	162 mg subcutaneous once weekly for 48 weeks	Neutropenia, rash thrombocytopenia, hepatic injury, anaphylaxis, and urticarial

^a^ Not terminated

#### Bosentan

Bosentan (Tracleer) is a dual ET-1R inhibitor used in the PAH treatment. Bosentan which blocks both ET_A_ and ET_B_ receptors is thought to exert a greater effect on ET_A_R, causing a total decrease in pulmonary vascular resistance [97]. According to Rezus et al., this medication can prevent the new digital ulcers in SSc patients. Via reducing the burden of Raynaud’s and digital ulcer-related symptoms, bosentan may indirectly impact the functionality and quality of life in the SSc patients. Nonetheless, improved outcomes were observed in the SSc patients and decreased symptom burden at baseline [98]. Some phase IV clinical trials (NCT02798055 and NCT01836263) are underway that aim to assess bosentan effects in SSc patients. The use of bosentan in the treatment of patients with SSc disorder can prevent the digital ulcers, a vascular event of SSc consistent. Its beneficial effect on PAH is related with connective tissue disease and vascular remodeling.

#### Imatinib

Imatinib is a specific tyrosine kinase receptor inhibitor that is used in the therapy of philadelphia chromosome-positive chronic myelogenous leukemia and inhibits the receptor tyrosine kinases for PDGF in SSc [99]. PDGF and TGF-β production through fibroblasts requires tyrosine kinase signaling events. Inhibition of tyrosine kinase might be a key signal modifier for the treatment of SSc. It has been proposed by the preliminary data that imatinib may be helpful in the treatment of SSc, as it may be useful in decreasing skin manifestations, PAH, and possibly, interstitial lung disease associated with the SSc disease. However, it did not show efficacy in the second phase of the clinical trial in SSc disease and the high dose of imatinib may lead to severe adverse events [100]. However, some clinical trials indicated that the safety and efficacy of imatinib mesylate for the cure of fibrosis in patients with SSc (NCT00613171).

#### Ambrisentan

Ambrisentan is an ETR inhibitor with ET_A_R selectivity being developed for the targeted treatment of PAH and SSc patients (NCT02290613) [101]. The evaluation of ambrisentan was performed in two large phases III randomized controlled trials in ARIES-1 and ARIES-2 for the PAH and SSc treatment. Ambrisentan dosed at either 5 or 10mg orally once per day (NCT01051960).

#### Sirolimus

Sirolimus, known also as rapamycin, is a macrolide compound that is used to prevent solid organ transplant rejection and treat fibrotic lung diseases (NCT01079143) including SSc patients. It inhibits the activation and proliferation of T and B lymphocytes by decreasing their sensitivity to IL-2 through inhibition of mTOR [102]. As PI3K/Akt/mTOR/S6K1 signaling is needed for ATP-induced proliferation in fibroblasts of adventitial tissue, it is supported that mTOR is main to modulating proliferation of fibroblast [103]. Sirolimus has anti-inflammatory and anti-fibrotic effects in idiopathic pulmonary fibrosis (IPF) [96]. Multiple clinical trials in phase II are underway for a more precise examination of possible relations between sirolimus and SSc (NCT03365869). 

#### MMI-0100

MMI-0100 is supposed to be a first-class inhibitor of MAPKAP kinase 2 (MK2), a downstream kinase within the TGF-β pathway. MK2 is known as the mediator of fibrosis and inflammation. The experimental therapy has shown its effectiveness for the treatment of fibrosis and inflammation in many animal models. These models involve cardiac, vascular, pulmonary inflammation and fibrosis, as well as the prophylaxis of scar and the construction of adhesion in acute surgical models [104]. The cell-permeant peptide MMI-0100 by MK2 inhibition can alleviate acute fibrotic injury [105]. Meng et al. examined the influence of MMI-0100 inhibition of the MK2 cascade in reducing fibrosis. According to these researchers, long-term treatment with MMI-0100 could inhibit α-SMA expression, block fibroblast differentiation, diminish extracellular collagen deposition, and alleviate cardiac fibrosis in the cMyBP-C heart animal models [106].

#### Salirasib

Salirasib is a salicylic acid derivative that inhibits neoplastic activation. Dislodging all Ras isoforms from their membrane-anchoring sites, salirasib inhibits Ras protein activation that mediates differentiation, proliferation, fibrosis and senescence of cells. Ras signaling is considered to be abnormally activated in one third of human cancers [107], autoimmune diseases such as multiple sclerosis (MS) [108] and probably in fibrotic diseases. Salirasib can inhibit the overexpression of P38 MAPK phosphorylation level, but it has a small effect on the JNK phosphorylation [109]. Several reports demonstrate that the enhanced activation level of MAPK pathway signaling is associated with fibrosis, and downregulation of MAPK pathway protein activation can meliorate the anti-fibrotic and anti-inflammatory process [56, 109, 110]. However, the outcomes of salirasib in combination with other medications, as therapeutic potential are suggested in fibrotic diseases such as SSc.

Accordingly, some studies have shown that salirasib alone and its combination with vitamin D (also referred to as calciferol) has anti-fibrotic effects on the liver, as it reduces main fibrotic marker expression such as collagen type I and tissue inhibitor of metalloproteinases-1 (TIMP-1) and, doing so, prevents the development of liver fibrosis [63]. It also can be used as a potential therapeutic medication for fibrotic diseases including SSc and IPF.

## Conclusions and future perspectives

The function of ET-1 and Ras in the pathogenesis of the dermis, lungs, or other organs fibrosis in SSc and other fibrotic diseases has been explored in several clinical and experimental studies. The potential merits of ET-1and Ras/ERK inhibitors in the vasculopathy and fibrosis in SSc and other fibrotic diseases have been discovered in recent clinical analyses; however, further thorough investigations and clinical studies concerning the assessment of the efficacy of the combination therapy of ET-1with Ras/ERK antagonists and other agents are awaited to yield reliable findings.

## Abbreviations

SSc: Systemic sclerosis
pDCs: Plasmacytoid dendritic cells
FN: Fibronectin
α-SMA: α-Smooth muscle actin
MMP-1: Matrix metalloproteinase
TGF-β: Transforming growth factor-β
ET-1: Endothelin-1
ECM: Extracellular matrix
CTGF: Connective tissue growth factor
IL: Interleukin
VWF: Von willebrand factor
HIF-1: Hypoxia-inducible factor-1
Ap-1: Activator protein 1
NF-κB: Nuclear factor kappa B
FOXO1: Forkhead-box protein O1
PMN: Polymorphonuclear
GPCRs: G-protein-coupled receptors
SMCs: Smooth muscle cells
NO: Nitric oxide
PAH: Pulmonary arterial hypertension
MVECs: Micro-vascular endothelial cells
EndMT: Endothelial-mesenchymal transition
PLCβ: Phospholipase Cβ
PIP2: Phosphatidylinositol-4, 5-bisphosphate
DAG: Diacylglycerol
IP3: Inositol triphosphate
ER: Endoplasmic reticulum
PKC: Protein kinase C
ALK5: Activin receptor-like kinase 5
NAb: Neutralizing antibodies
TCR: T cell receptors
MHC: Major histocompatibility complex
SLE: Systemic lupus erythematosus
EMT: Epithelial mesenchymal transition
Egr-1: Early growth response protein-1
FGF: Fibroblast growth factor
GEFs: Guanine nucleotide exchange factors
gp130: Glycoprotein 130
IL-6R: IL-6 receptor
IPF: Idiopathic pulmonary fibrosis
MS: Multiple sclerosis
